# Abdominal manifestations of extranodal lymphoma: pictorial essay*

**DOI:** 10.1590/0100-3984.2015.0201

**Published:** 2016

**Authors:** Laís Fajardo, Guilherme de Araujo Ramin, Thiago José Penachim, Daniel Lahan Martins, Patrícia Prando Cardia, Adilson Prando

**Affiliations:** 1 Physician in the Program for Continuing Education in Radiology and Diagnostic Imaging at the Centro Radiológico Campinas/Hospital Vera Cruz, Campinas, SP, Brazil; 2 Physician in the Program for Continuing Education in Radiology and Diagnostic Imaging at the Hospital e Maternidade Celso Pierro - Pontifícia Universidade Católica de Campinas (PUC Campinas), Campinas, SP, Brazil; 3 Full Member of the Colégio Brasileiro de Radiologia e Diagnóstico por Imagem (CBR); MD, Radiologist at the Centro Radiológico Campinas/Hospital Vera Cruz, at the Hospital e Maternidade Celso Pierro - Pontifícia Universidade Católica de Campinas (PUC Campinas), and at the Hospital de Clínicas da Universidade Estadual de Campinas (Unicamp), Campinas, SP, Brazil; 4 Full Member of the Colégio Brasileiro de Radiologia e Diagnóstico por Imagem (CBR); MD, Radiologist at the Centro Radiológico Campinas/Hospital Vera Cruz and at the Hospital e Maternidade Celso Pierro - Pontifícia Universidade Católica de Campinas (PUC Campinas), Campinas, SP, Brazil; 5 Full Member of the Colégio Brasileiro de Radiologia e Diagnóstico por Imagem (CBR); MD, Radiologist and Head of the Department of Radiology and Diagnostic Imaging at the Centro Radiológico Campinas/Hospital Vera Cruz, Campinas, SP, Brazil

**Keywords:** Lymphoma, Abdomen, Tomography, X-ray computed

## Abstract

In the appropriate clinical setting, certain aspects of extranodal abdominal lymphoma, as revealed by current cross-sectional imaging techniques, should be considered potentially diagnostic and can hasten the diagnosis. In addition, diagnostic imaging in the context of biopsy-proven lymphoma can accurately stage the disease for its appropriate treatment. The purpose of this article was to illustrate the various imaging aspects of extranodal lymphoma in the abdomen.

## Abstract (pt)

O linfoma extranodal abdominal tem um espectro de manifestações radiológicas que, no contexto clínico adequado, permite que o radiologista, ao considerar corretamente esta hipótese, otimize o seu diagnóstico e sua terapia. Além disso, depois de comprovado por biópsia, o linfoma pode ser estadiado e a resposta ao tratamento, avaliada pelos métodos de imagem. O presente ensaio iconográfico tem como objetivo ilustrar os diferentes achados por imagem do acometimento extranodal no linfoma abdominal.

## INTRODUCTION

Extranodal involvement occurs in 40% of patients with lymphoma^(1)^, representing lymphoproliferative disease in various tissues of the lymph nodes, thymus, and tonsils. The exception is the spleen, where it is classified as extranodal in non-Hodgkin lymphoma (NHL) and nodal in Hodgkin lymphoma^(2)^. Lymphomas are classified as primary extranodal when there is no nodal involvement or when there is minimal lymph node involvement and the extranodal component is dominant^(2,3)^. Secondary involvement, such as advanced disease, is significantly more common^1,4)^. The extranodal form is more common in NHL, occurring in 25% of cases. On computed tomography (CT), a lymphoma usually presents with slightly less density than does the parenchyma of the organ involved^(2)^. It often shows little contrast uptake^(2)^. Heterogeneous enhancement can be seen in large masses, due to areas of central necrosis. Unless previously treated, lymphomas do not typically present calcifications^(2)^.

## SPLEEN

**Figure 1:**
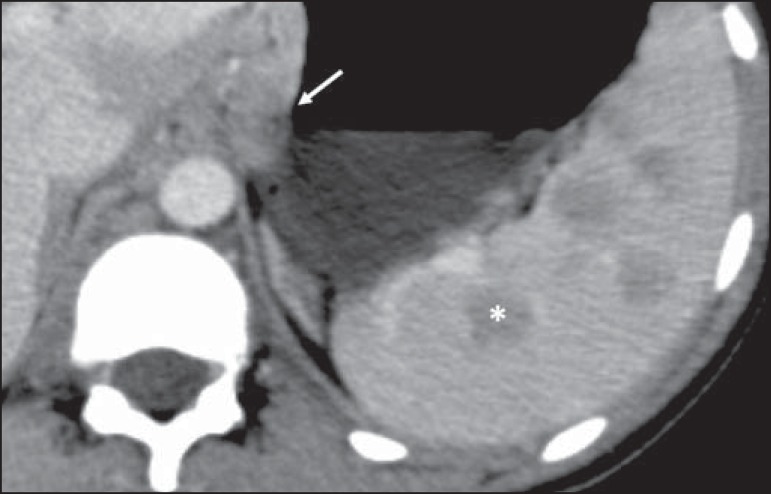
Multinodular splenic involvement by lymphoma. Contrast-enhanced axial CT of the abdomen, showing multiple hypovascularized nodules in the spleen (asterisk) and perigastric lymph node involvement (arrow).

Splenic involvement occurs in 30-40% of patients with lymphoma^(5)^. Primary splenic lymphoma is rare. Diffuse infiltration is the most common form of presentation, often resulting in homogeneous splenomegaly, although the spleen can also be of normal size. In such cases, functional imaging methods, such as FDG-PET/CT, facilitate the diagnosis^(1)^. Solitary masses and multifocal lymphomatous nodules are hypointense on CT, with little contrast uptake (Figure 1). One should distinguish them from splenic infarcts, as well as from fungal microabscesses.

## LIVER

**Figure 2:**
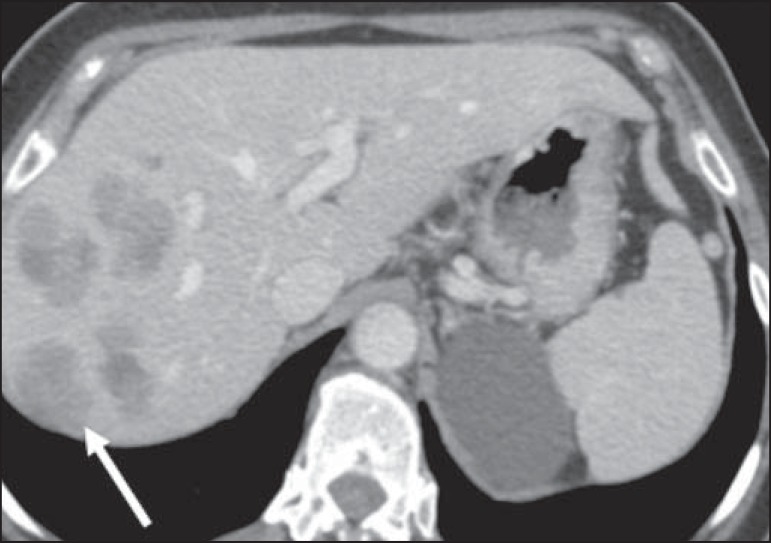
Hepatic lymphoma. Axial CT of the abdomen, showing multiple nodules, of varying sizes, in the liver, most of the nodules being seen to be hypovascularized after the injection of contrast medium (arrow).

Primary hepatic lymphoma is rare^(2)^. Diffuse infiltration is the most common pattern and can be easily overlooked because of its homogeneous aspect^(5)^. The nodular pattern is seen in only 10% of cases. Lymphomatous multifocal masses and miliary lesions are similar to metastatic disease and microabscesses. The differentiation is based on the relatively homogeneous aspect of the lymphoma (Figure 2), and magnetic resonance imaging can help. There can be periportal lymphomatous infiltration extending from the porta hepatis.

## GALLBLADDER

**Figure 3:**
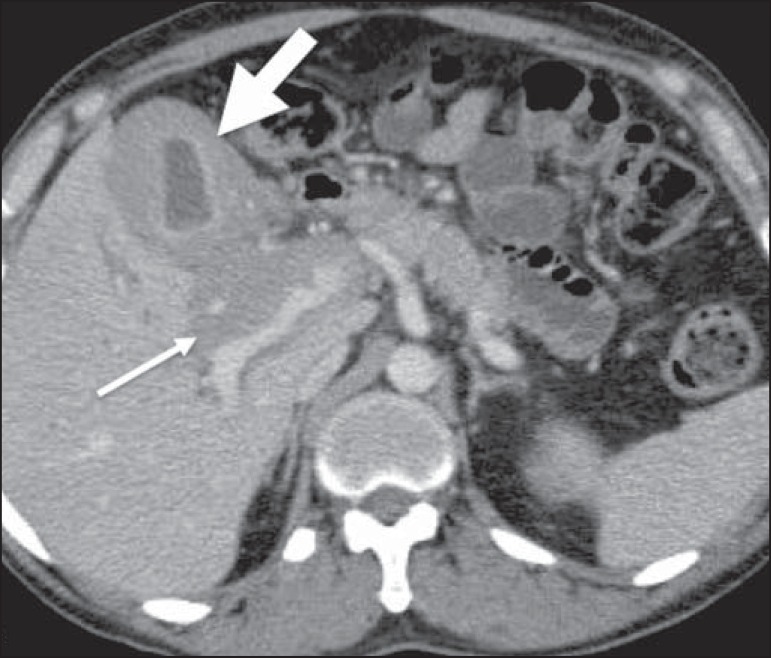
Gallbladder involvement by lymphoma. Axial CT of the abdomen, showing marked parietal thickening of the gallbladder (broad arrow), together with periportal lymphomatous infiltration (narrow arrow).

Primary lymphoma of the gallbladder is very rare. It manifests as focal or diffuse parietal thickening (Figure 3). When occurring in isolation, lymphoma of the gallbladder can be difficult to differentiate from inflammatory cholecystic disease.

## PANCREAS

**Figure 4:**
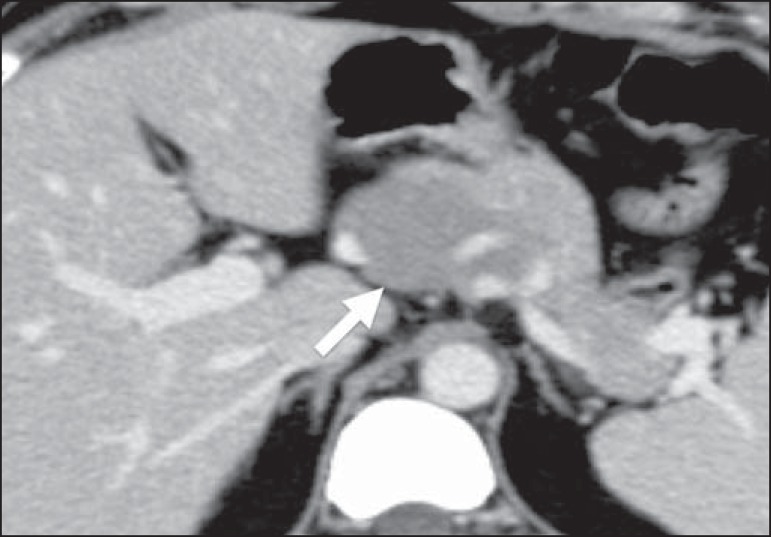
Pancreatic lymphoma. Contrast-enhanced axial CT of the abdomen, showing a well-defined, homogeneous, hypovascularized focal mass at the head of the pancreas (arrow), altering the path of the adjacent blood vessels.

**Figure 5:**
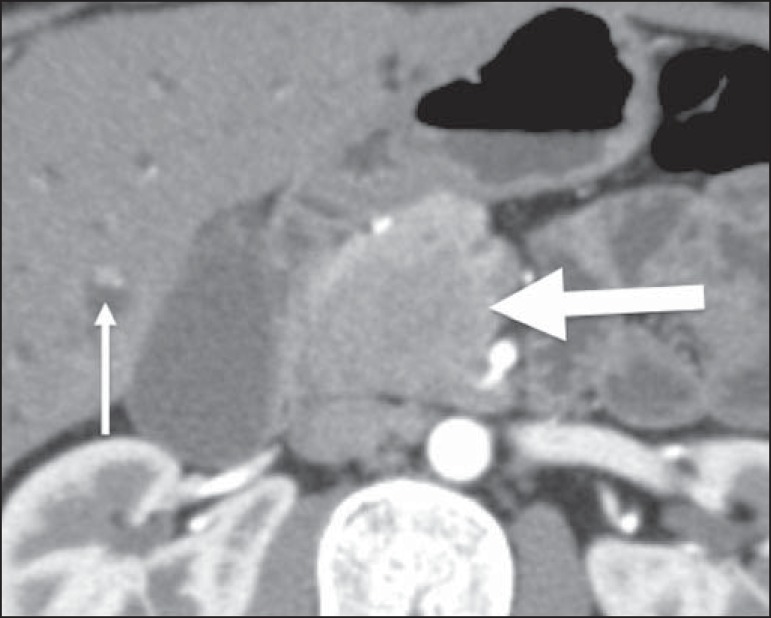
Pancreatic lymphoma. Contrast-enhanced CT of the abdomen, showing an expansive, solid, hypovascularized lesion at the head of the pancreas (broad arrow). The lesion promotes stenosis of the biliary tract in its intrapancreatic portion, with upstream dilatation, including the intrahepatic segments (narrow arrow).

The pancreas is affected in approximately 30% of cases of NHL, usually because it is contiguous to the lymph node involvement^(1,2)^. There are two distinct morphological patterns: a circumscribed focal mass (Figure 4); and diffuse enlargement due to infiltration. It can resemble acute pancreatitis, because of a diffuse decrease in contrast^(5)^. There is typically no ductal dilatation or significant parenchymal atrophy, even when there is invasion of the pancreatic duct, which supports the hypothesis of lymphoma instead of adenocarcinoma, the main differential diagnosis^(5)^. When it affects the cranial and intrapancreatic portions of the common bile duct, it can cause bile duct dilatation (Figure 5).

Other findings that favor the diagnosis of lymphoma are homogeneous density of the lesion and of the highlighting, together with the absence of vascular invasion and of calcifications, as well as the involvement of mesenteric and retroperitoneal lymph nodes.

## STOMACH

**Figure 6:**
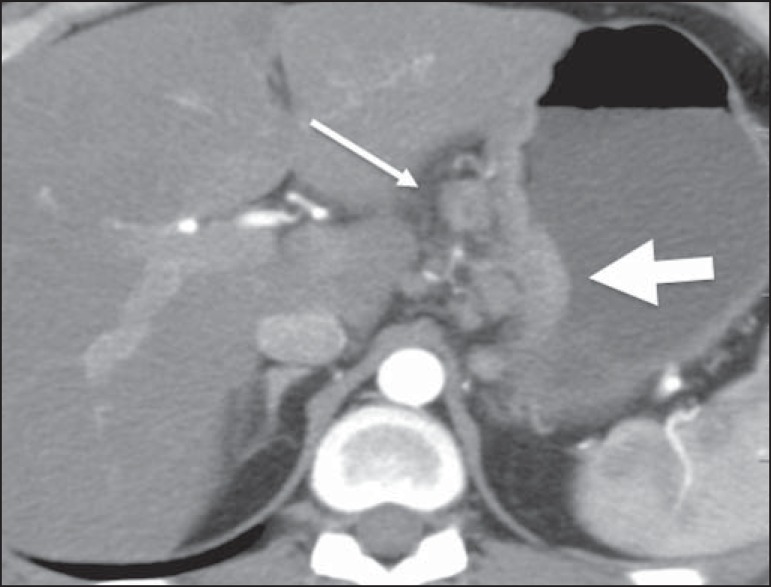
Gastric lymphoma. Axial CT of the abdomen, showing thickening of the wall of the lesser curvature of the stomach (broad arrow), together with regional lymph node involvement (narrow arrow).

**Figure 7:**
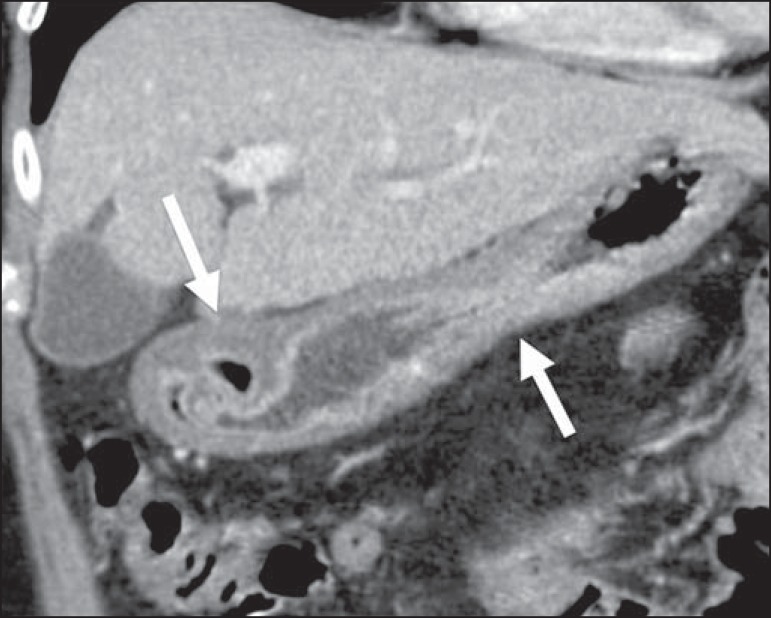
Gastric lymphoma. Contrast-enhanced CT of the abdomen, in coronal reconstruction, showing diffuse circumferential thickening of the walls of the gastric body and antrum (arrows).

The gastrointestinal tract (GIT) is the most common abdominal site of primary extranodal lymphoma, the stomach being the segment most often affected^(4)^. It is known that *Helicobacter pylori* plays a role in the etiopathogenesis of B-cell mucosa-associated lymphoid tissue type lymphoma, which accounts for 50-70% of primary gastric lymphomas. The most common imaging findings are focal thickening of the gastric wall (Figure 6), usually to a thickness greater than 3 cm, or circumferential parietal involvement (Figure 7). Primary gastric lymphoma can also manifest as a polypoid growth. A reduction in the size of the lumen of the gastric chamber is uncommon, as is upper gastrointestinal bleeding. Although lymphoma and adenocarcinoma can both be accompanied by lymphadenopathy, lymph node enlargement is more extensive in lymphoma.

## SMALL INTESTINE

**Figure 8:**
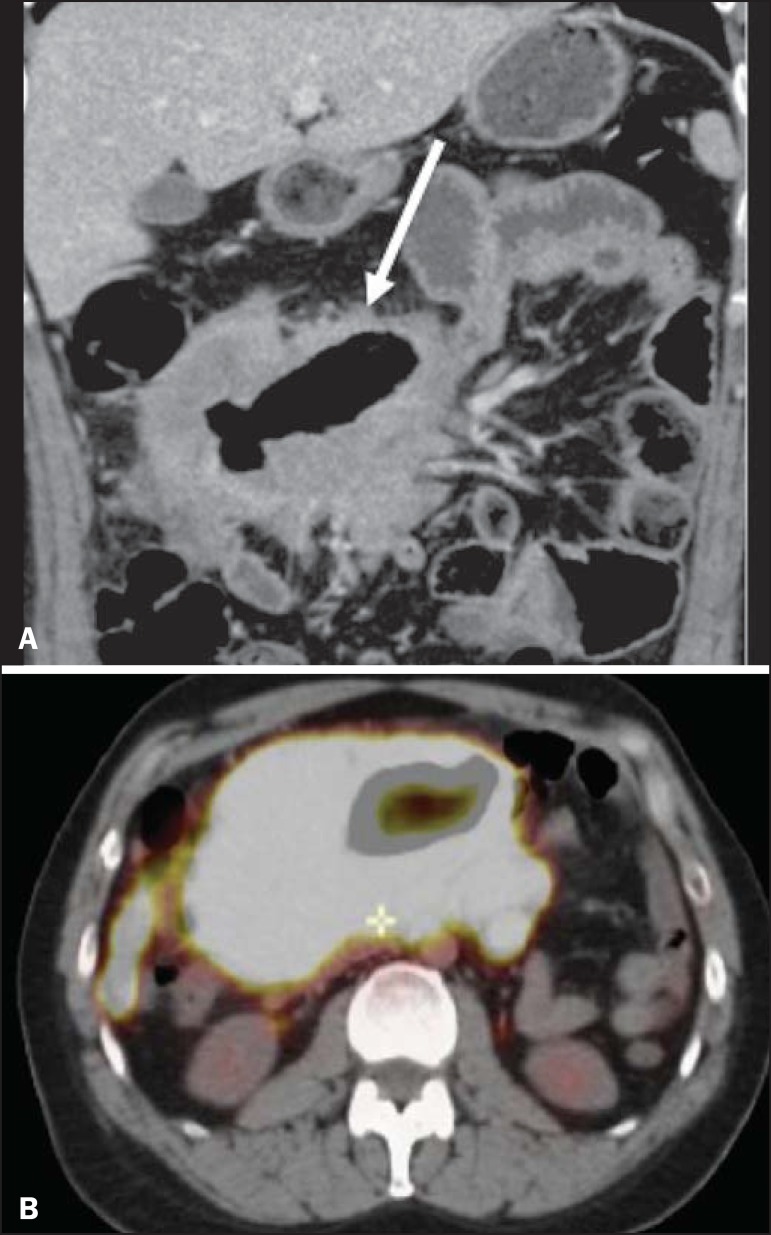
Primary NHL of the ileum. **A:** Coronal CT of the abdomen, in coronal reconstruction, showing marked, concentric parietal thickening, associated with aneurysmal dilatation of the ileal loop (arrow). **B:** PET/CT scan showing hypermetabolism at the site of the tumor.

The small intestine is the second most common GIT site affected by lymphoma, the distal ileum being the segment most often affected. Primary lymphoma accounts for over 50% of cases. Lymphoma of the small intestine can present as a polypoid mass, multiple nodules, or (asymmetric or concentric) thickening of the intestinal wall. It typically promotes aneurysmal dilation of the intestinal loop (Figure 8). The primary differential diagnoses are adenocarcinoma, Crohn's disease, and other inflammatory processes.

**Figure 9:**
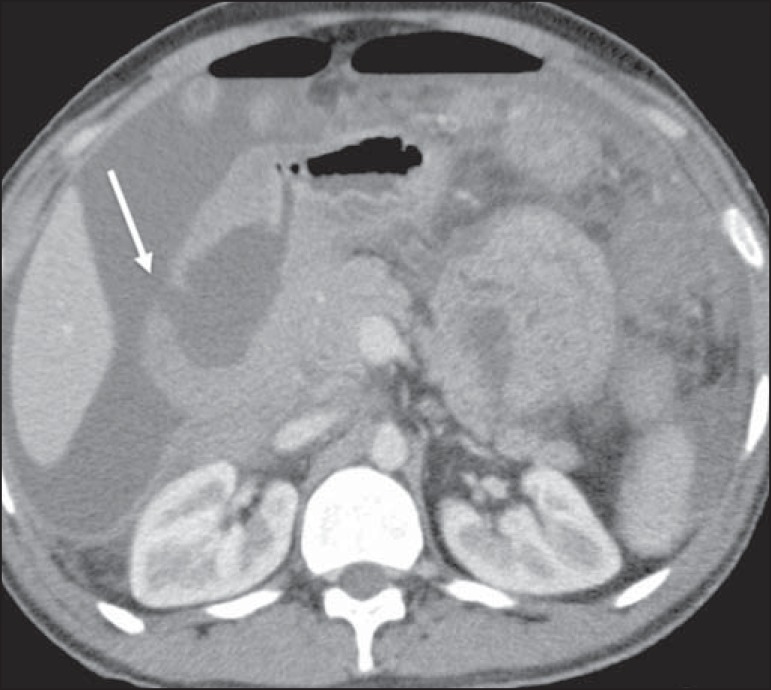
Intestinal perforation related to the treatment of lymphoma. Contrastenhanced axial CT of the abdomen, showing high-grade lymphoma of the duodenum in a patient undergoing chemotherapy, with tumor regression followed by intestinal perforation (arrow).

One important clinical condition that can occur in the context of GIT lymphoma is perforation as the tumor recedes during chemotherapy, which is mainly associated with lymphomas that invade the intestinal wall and respond well to chemotherapy (Figure 9).

## COLON

**Figure 10:**
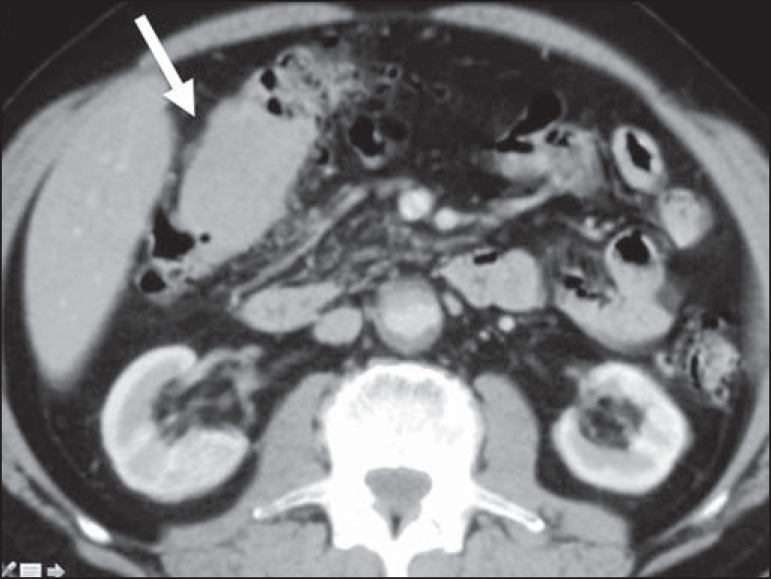
Lymphoma of the colon. Contrast-enhanced axial CT of the abdomen, showing a hypovascularized infiltrative parietal lesion in the hepatic flexure of the colon, with thickening of the local haustra (arrow).

The colon is the third place most common site affected by GIT lymphoma. The cecum and rectum are the sites most often involved^(1)^. Lymphoma of the colon can manifest as a bulky polypoid growth, which is the most common pattern and can cause invaginations. In contrast-enhanced imaging studies, it can also present as an infiltrative mass, thickening of the haustra (Figure 10), and mucosal nodularity. As opposed to colorectal adenocarcinoma, which is the main differential diagnosis, lymphoma affects long segments and, although it can narrow the lumen, rarely creates an obstruction^(1)^.

## ADRENAL GLANDS

**Figure 11:**
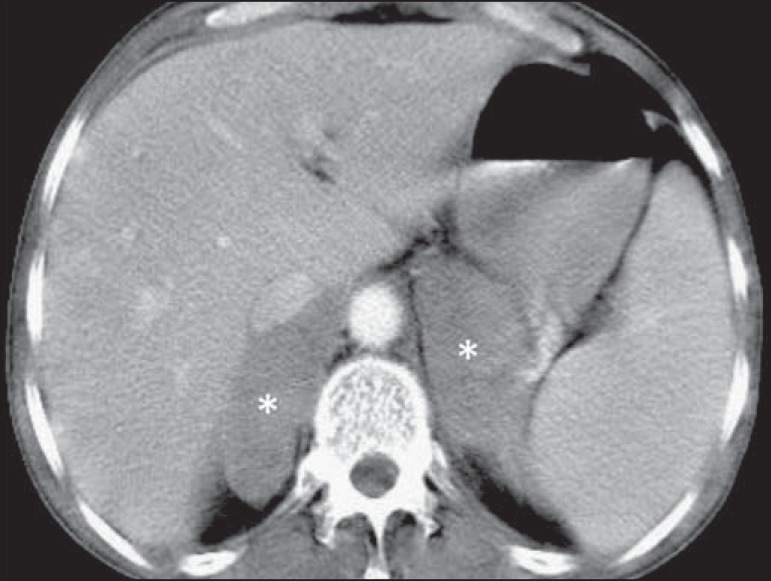
Lymphoma of the adrenal gland. Contrast-enhanced axial CT of the abdomen, showing homogeneous, hypovascularized nodular masses in the adrenal glands (asterisks).

The adrenal glands are involved in about 4% of all cases of NHL^(1,5)^, bilaterally in 50% of those cases. The morphological patterns include well-defined homogeneous masses (Figure 11), small nodules, and a diffuse increase in the size of the glands. Non-lymphomatous bilateral adrenal hyperplasia can coexist with lymphoma and must be differentiated from neoplastic involvement, mainly by FDG-PET/CT^(5)^.

## KIDNEYS

**Figure 12:**
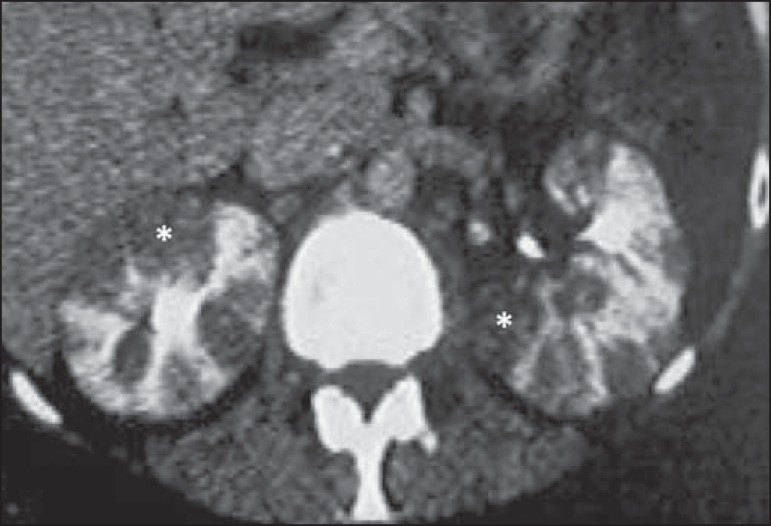
Primary renal lymphoma. Contrast-enhanced CT of the abdomen showing multiple, bilateral renal nodules with hypoattenuation in relation to the renal parenchyma (asterisks).

**Figure 13:**
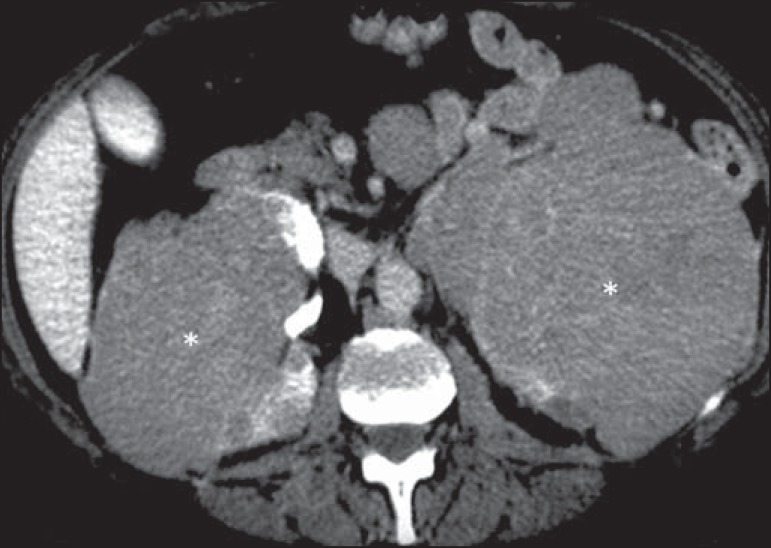
Renal lymphoma. Axial CT of the abdomen, without contrast, showing bilateral nephromegaly without alteration in the shape of the kidneys (broad arrows). Iodinated contrast was not administered, because the patient had renal failure. Not also the splenomegaly (asterisk) and lymph node involvement (narrow arrow).

**Figure 14:**
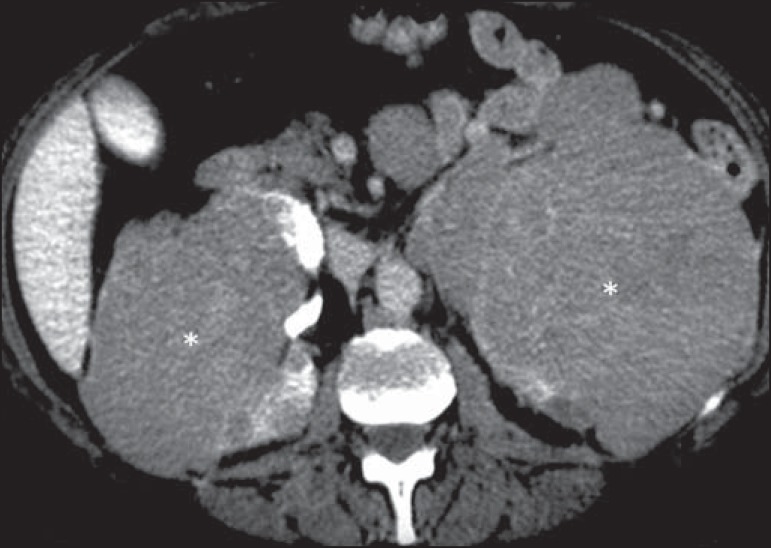
Primary renal lymphoma. Contrast-enhanced axial CT of the abdomen showing discretely heterogenous, low-attenuation, bulky masses (asterisks) infiltrating the kidneys and the retroperitoneal space.

**Figure 15:**
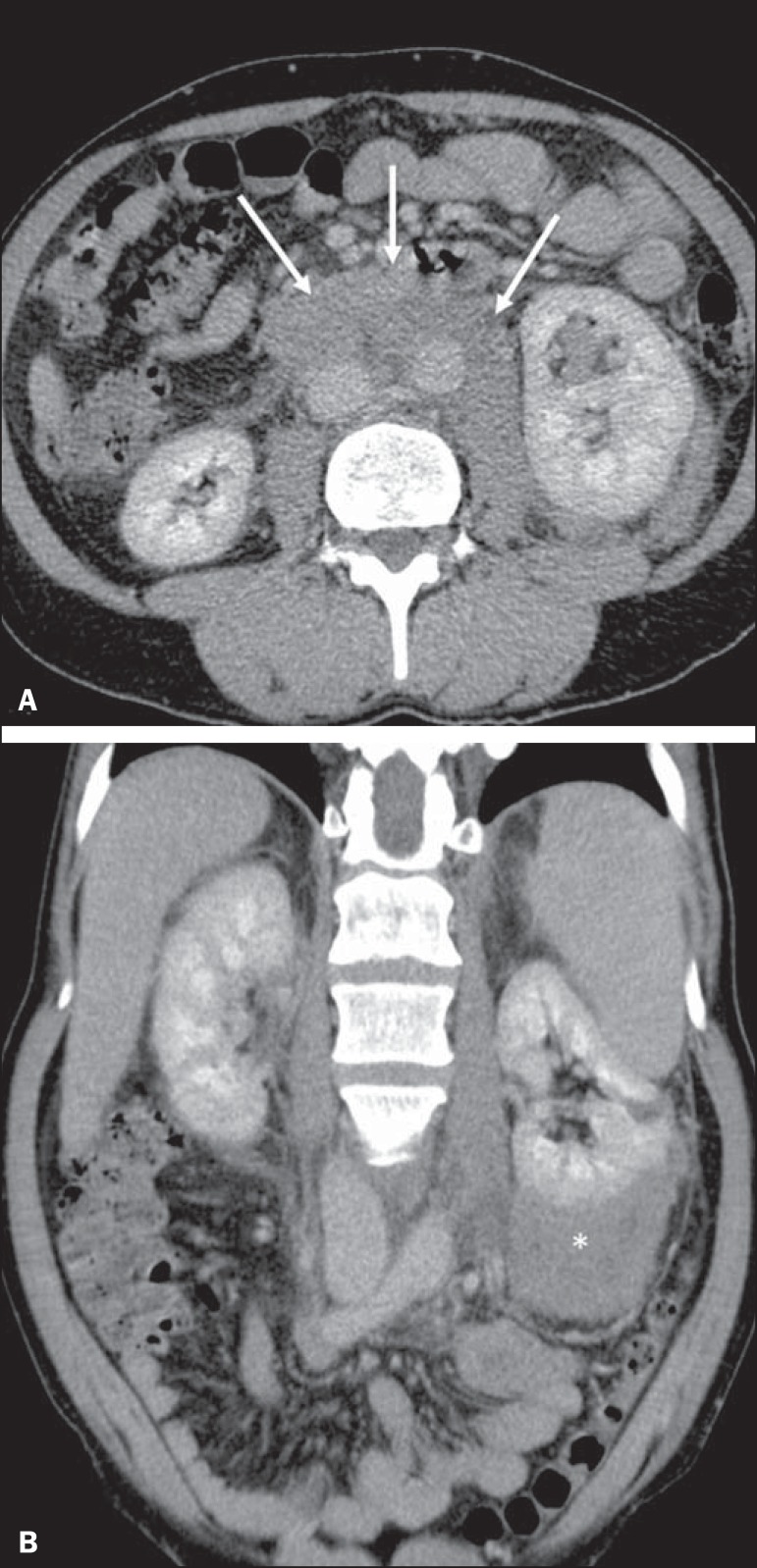
Contiguous renal invasion in retroperitoneal disease. Contrast-enhanced CT of the abdomen, in the axial plane (**A**) and in coronal reconstruction (**B**), showing extensive retroperitoneal lymph node involvement (arrows) and a hypovascularized mass encompassing the inferior pole of the left kidney (asterisk). The symmetrical pattern of contrast uptake indicates that there was no compression of the parenchyma or impairment of renal function in the affected kidney.

**Figure 16:**
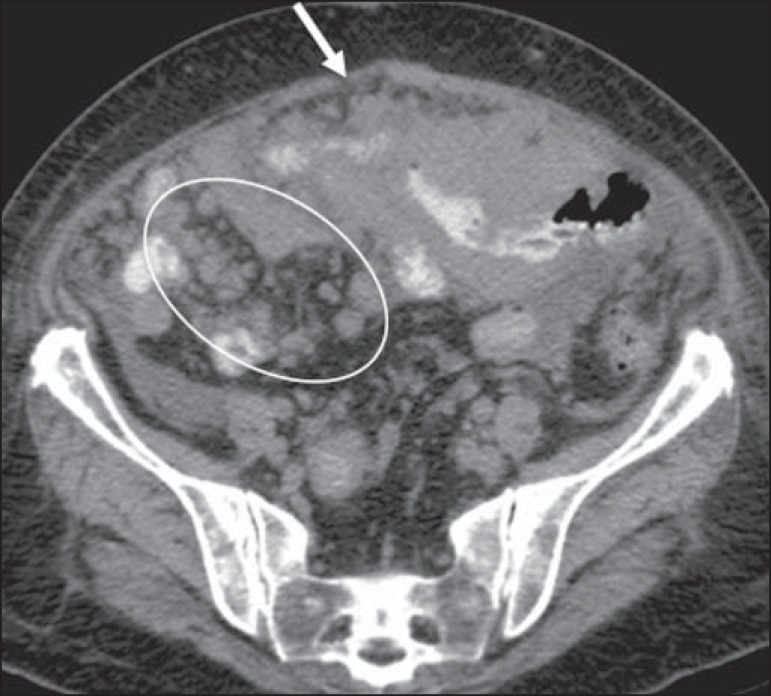
Renal lymphoma. Contrast-enhanced CT of the abdomen, in the axial plane (**A**) and in coronal reconstruction (**B**), showing the perirenal form of presentation of lymphoma. Hypovascularized left perirenal tumor infiltrating the parenchyma (arrows).

In advanced cases of nodal lymphoma, renal involvement is relatively common. Primary renal lymphoma is extremely rare. In 60% of cases, it manifests as multiple, bilateral, homogeneous nodules, with discrete and sometimes progressive impregnation^(2)^ (Figure 12). Diffuse lymphomatous infiltration occurs in 20% of cases, preserves the shape of the kidney, and is typically bilateral (Figure 13). Renal masses occur in 10-20% of cases^(2)^, can mimic papillary renal cell carcinoma due to the pattern of homogeneous hypovascular impregnation (Figure 14). Contiguous renal invasion in retroperitoneal disease is common and typically manifests as a bulky mass that envelopes but does not obstruct the renal vessels. It usually invades the hilum and displaces the affected kidney (Figure 15). Perirenal involvement that is not secondary to retroperitoneal disease is uncommon. It manifests as homogeneous tissue with density of the soft parts encompassing the renal parenchyma, without causing a significant loss of function (Figure 16). In milder cases, the findings can be limited to thickening of the renal fascia or nodules in the perirenal space. The differential diagnoses include sarcoma of the renal capsule and metastases, as well as benign conditions such as perirenal hematoma, retroperitoneal fibrosis, amyloidosis, and extramedullary hematopoiesis.

## PERITONEUM AND MESENTERY

**Figure 17:**
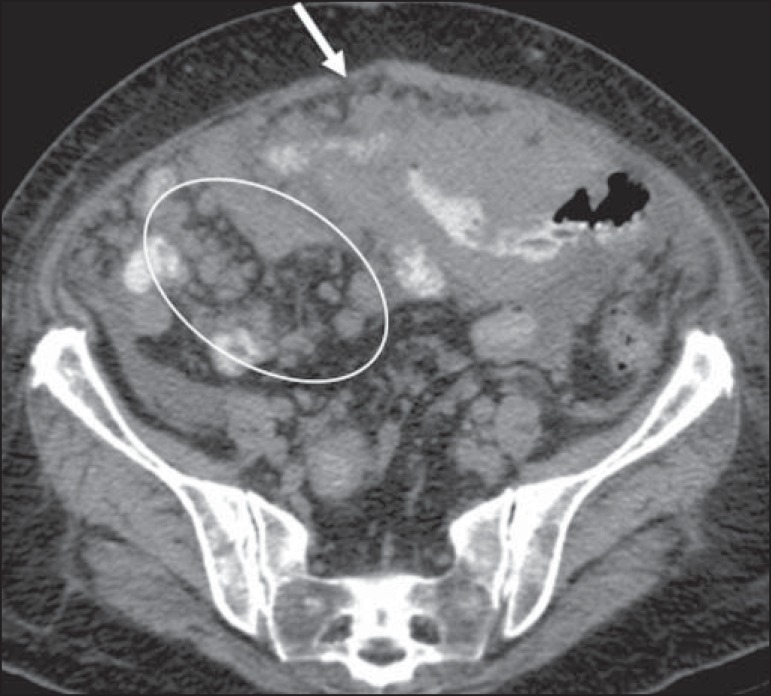
Peritoneal lymphomatosis. Axial CT of the abdomen, showing increased mesenteric density and mesenteric nodules (elliptical contour) and peritoneal nodular thickening (arrow), secondary to dissemination of lymphoproliferative disease.

Lymphoma is the most common cause of masses and nodules in the mesenteric lymph nodes, which are most often associated with GIT lymphoma. Such changes are radiologically indistinguishable from those occurring in peritoneal carcinomatosis^(1)^. Increased mesenteric density is most commonly seen after treatment^(5)^. The patterns of involvement include peritoneal nodules (Figure 17) and a diffuse infiltrative mass. It results in hardening/thickening of the leaflets of the mesentery and an omental cake aspect. Exudative ascites with high attenuation can occur^(1)^.

## IMMUNOCOMPROMISED PATIENTS

There is a high incidence of NHL in immunocompromised transplant recipients^(6)^. It is a serious and relatively common complication, occurring in 2-5% of such patients, and is particularly common after treatment with cyclosporine^(2,5)^. Its incidence peaks in the first year after transplantation, and it can affect virtually any organ. Isolated lymph node involvement is less common, occurring in only 20% of cases^(7)^.

## CONCLUSION

Extranodal lymphoma can simulate other neoplasms or inflammatory conditions. The radiologist plays a crucial role in the noninvasive management of the disease. In addition to raising suspicion of the diagnosis, the role of the radiologist includes obtaining material via percutaneous biopsy, facilitating the staging, assessing the response to or complications of treatment, and identifying recurrence or relapse.
